# Lanostane Triterpenoid Metabolites from a *Penares* sp. Marine Sponge Protect Neuro-2a Cells against Paraquat Neurotoxicity

**DOI:** 10.3390/molecules25225397

**Published:** 2020-11-18

**Authors:** Ekaterina A. Yurchenko, Sophia A. Kolesnikova, Ekaterina G. Lyakhova, Ekaterina S. Menchinskaya, Evgeny A. Pislyagin, Ekaterina A. Chingizova, Dmitry L. Aminin

**Affiliations:** 1 Laboratory of Bioassays and Mechanism of Action of Biologically Active Substances, G.B. Elyakov Pacific Institute of Bioorganic Chemistry, prosp. 100 let Vladivostoku 159, 690022 Vladivostok, Russia; ekaterinamenchinskaya@gmail.com (E.S.M.); pislyagin@hotmail.com (E.A.P.); martyyas@mail.ru (E.A.C.); daminin@piboc.dvo.ru (D.L.A.); 2 Laboratory of Marine Natural Products Chemistry, G.B. Elyakov Pacific Institute of Bioorganic Chemistry, prosp. 100 let Vladivostoku 159, 690022 Vladivostok, Russia; sovin81@inbox.ru (S.A.K.); elyakhova@inbox.ru (E.G.L.); 3 Department of Biomedical Science and Environmental Biology, Kaohsiung Medical University, No.100, Shin-Chuan 1st Road, Sanmin Dist., Kaohsiung City 80708, Taiwan

**Keywords:** paraquat, reactive oxygen species, membrane mitochondrial potential, neurite outgrowth, Hsp70, neuroprotection, Parkinson’s disease, *Penares*, lanostane triterpenoids

## Abstract

The results of an investigation of the protective effects of five lanostane triterpenoids: 3*β*-acetoxy-7*β*,8*β*-epoxy-5*α*-lanost-24-en-30,9*α*-olide (**1**), 3*β*-hydroxy-7*β*,8*β*-epoxy-5*α*-lanost-24-en- 30,9*α*-olide (**2**), 29-*nor*-penasterone (**3**), penasterone (**4**), and acetylpenasterol (**5**), from a marine sponge, *Penares* sp., against paraquat-induced neuroblastoma Neuro-2a cell damage, are described. The influence of all compounds on viability of the Neuro-2a cells treated with paraquat (PQ) was studied with MTT and fluorescein diacetate assays as well as propidium iodide straining. 1,1-Diphenyl-2-picrylhydrazyl (DPPH) radical scavenging activity of the compounds as well as their influence on reactive oxygen species (ROS) level and mitochondrial membrane potential in PQ-treated neuronal cells were analyzed. Finally, the effect of the compounds on intracellular level of heat shock protein 70 kDa (Hsp70) and neurite outgrowth in PQ-treated Neuro-2a cells were studied. Studied triterpenoids demonstrated protective effects against PQ-induced neurotoxicity associated with the ability to reduce ROS intracellular level and diminish mitochondrial dysfunction. Acetylpenasterol (**5**), as a more promising neuroprotective compound, significantly increased the viability of Neuro-2a cells incubated with PQ as well as decreased intracellular ROS level in these cells. Moreover, acetylpenasterol induced Hsp70 expression in PQ-treated cells. It was also shown to inhibit PQ-induced neurite loss and recovered the number of neurite-bearing cells. The relationship between neuroprotective activity of the investigated compounds **1**–**5** and their chemical structure was also discussed.

## 1. Introduction

Environmental toxins such as herbicides and pesticides and their damaged effects on humans are major problems for countries where agriculture is widespread. Paraquat (*N*,*N*-dimethyl-4,4-bipyridinium dichloride, PQ) is a herbicide belonging to the chemical class of bipyridyl quaternary ammonium herbicides [1]. PQ poisoning is one of the leading intoxications worldwide, with no effective antidote and treatment protocol. However, this herbicide is still used in over 120 countries. As a result, cases of human deaths from the toxic effects of PQ are not rare [2].

The mechanism of PQ lethal pneumo- and neurotoxicity suggests the accumulation in alveolar cells, neurons, and astroglia and the resulting oxidative stress events. In addition to the formation of reactive oxygen species (ROS), PQ-induced toxicity has been associated with the induction of endoplasmic reticulum, apoptosis, mitochondrial damage, and inflammation, as evidenced by the regulation of various protein/signaling pathways [3]. The neurotoxicity of PQ is well studied and described as mediated by the induction of a redox cycling with cellular diaphorases (enzymes that transfer one electron from NADPH), such as NADPH-oxidases and nitric oxide synthase, with subsequent production of ROS [4]. Moreover, it was reported that PQ is a specific inhibitor of neurite outgrowth, since it downregulates the genes of the “Cellular Growth and Proliferation” group [5]. Thus, exposure to PQ has led to an increased risk of neurodegenerative disorders, including Parkinson’s disease (PD) [6]. Recently, it was estimated that as a result of agricultural pesticide application, airborne PQ contributes nearly 21% and 24% respectively, to the PD prevalence rates among the age groups of 70–79 and ≥80 years in Taiwan [7].

For this reason, PQ is commonly used in in vitro and in vivo experiments as an inducer of Parkinson’s disease symptoms [8,9], despite the fact that these in vitro PD models have some limitations [10]. Therefore, the study of paraquat in relation to Parkinson’s disease is warranted [11]. The cellular mechanisms of protection against PQ toxicity include activation of antioxidant pathways and autophagy as well as overexpression of a heat shock proteins’ machinery [12,13,14,15]. Thus, compounds that can trigger one or all of these strategies are of interest as cytoprotective agents.

Terpenoids, and especially triterpenoids, are the largest and the most diverse group of naturally occurring organic compounds. Some triterpenoids and their semi-synthetic derivatives were identified as potential therapeutics against neurodegenerative diseases [16]. For instance, two 11-methoxyl substituted triterpenoids mimengosides J and K from the fruits of *Buddleja lindleyana* were reported to be protective compounds against damage of SH-SY5Y cells induced by 1-methyl-4-phenylpyridinium ion (MPP+) [17]. It was published recently that ergosta-4,6,8(15),22-tetraen-3-one, 11*α*-ethoxy-*β*-boswellic and acetyl-11-keto-*β*-boswellic acids, mansumbin-13(17)-en-3,16-dione, and 3*α*-acetoxy-mansumbin-13(17)-en-16-one from *Populus euphratica* resins protected mouse hippocampal neuronal HT-22 cells from H_2_O_2_-induced oxidative stress injury. The last two compounds listed above, belonging to the structural group of octanortriterpenoids, also prevented glutamate-induced excitotoxicity in human neuroblastoma SH-SY5Y cells [18]. The lanostane triterpenoids from one of the most famous medicinal fungi, *Ganoderma lucidum,* and other *Ganoderma* mushrooms, have shown anti-neuroinflammatory, antioxidant, and cytoprotective activities in numerous studies [19,20,21,22]. Also, three new ergostane-type triterpenoids, antcamphorols G, I, and J from another medicinal mushroom *Antrodia camphorate,* showed significant ROS scavenging activities in high-glucose-induced human umbilical vein endothelial cells (HUVECs) [23].

**Figure 1:**
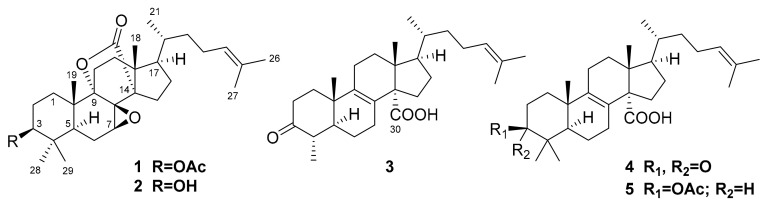
The structures of investigated compounds: 3*β*-acetoxy-7*β*,8*β*-epoxy-5*α*-lanost-24-en-30,9*α*-olide (**1**), 3*β*-hydroxy-7*β*,8*β*-epoxy-5*α*- lanost-24-en-30,9*α*-olide (**2**), 29-*nor*-penasterone (**3**), penasterone (**4**), and acetylpenasterol (**5**).

The lanostane triterpenoids, their demethylated derivatives, and steroids derived thereof from marine sponges of the genus *Penares* represent a particular group of natural products. The structural feature of these compounds is the presence of carboxy group, attached to C-14, or 30,9-lactone fragment in lanostane nucleus [24]. 3*β*-Acetoxy-7*β*,8*β*-epoxy-5*α*-lanost-24-en-30,9α-olide (**1**), 3*β*-hydroxy-7*β*,8*β*-epoxy-5*α*-lanost-24-en-30,9*α*-olide (**2**), and 29-*nor*-penasterone (**3**) were first isolated during the study on Vietnamese collection of *Penares* sp. marine sponge together with known penasterone (**4**), acetylpenasterol (**5**) (Figure 1) [25], penasterol, and a series of minor oxidized lanostane and *nor*-lanostane derivatives [24]. The cytotoxicities of isolated triterpenoids **1**–**5** against several tumor cell lines, including Ehrlich ascites carcinoma, human promyelocytic leukemia HL-60, and human cervical carcinoma HeLa, were measured, but none of the compounds showed significant effects [25]. However, penasterone (**4**) and acetylpenasterol (**5**) were reported as inhibitors of histamine release induced by anti-IgE [26].

Herein, we report the results on the investigation of the protective effects of compounds **1**–**5** against PQ-induced neuroblastoma Neuro-2a cell damage. For this purpose, the influence of compounds **1**–**5** on viability of the Neuro-2a cells treated with PQ were studied. DPPH radical scavenging activity of compounds as well as their influence on ROS level and mitochondrial membrane potential in PQ-treated neuronal cells were analyzed. Finally, we assessed whether the compounds affect intracellular level of heat shock protein 70 kDa (Hsp70) and neurite outgrowth in PQ-treated Neuro-2a cells.

Thus, the present work aimed to investigate various aspects of neuroprotective activity of five *Penares* sp. lanostane triterpenoids structurally related to penasterol.

## 2. Results

### 2.1. Cytotoxicity toward Neuro-2a Cells

The cytotoxic activity of five lanostane triterpenoid metabolites from *Penares* sp. sponge was evaluated by the MTT assay (Table 1). Only triterpene epoxylactone **1** was toxic to Neuro-2a cells with a half-maximal inhibitory concentration (IC_50_) of 85.7 μM. Compounds **3** and **5** caused the death of 37.4% and 45.6% of cells respectively, at a concentration of 100 μM. All compounds were nontoxic at a concentration of 10 μM, therefore this and lower concentrations were used for further investigations.

### 2.2. DPPH Radical Scavenging Activity

Ascorbic acid scavenged half of DPPH radicals at a concentration of 20.5 μM. All investigated compounds, **1**–**5**, did not demonstrate any DPPH-radical scavenging effect with a half-maximal effective concentration (EC_50_) more than 100 μM in this cell-free assay (Table 1).

### 2.3. Influence on PQ-Treated Neuro-2a Cell Viability

**Figure 2:**
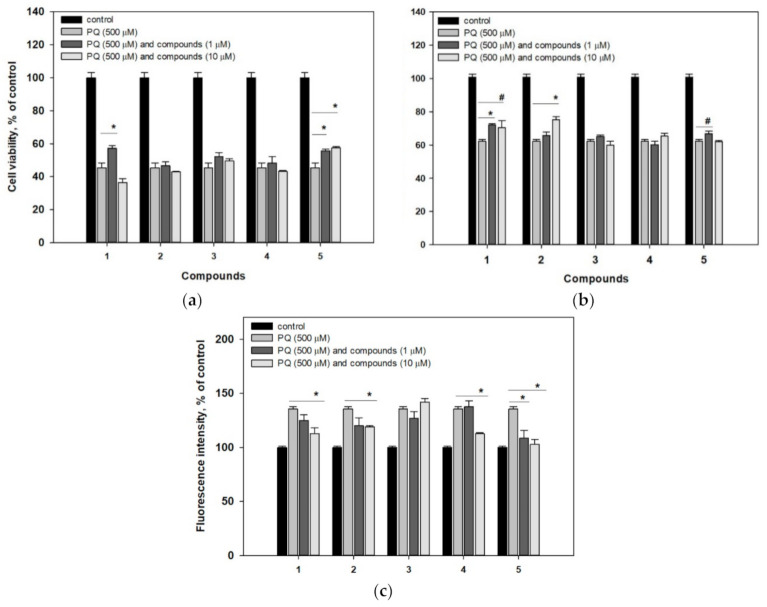
Influence of compounds **1**–**5** on PQ-treated Neuro-2a cell viability. (**a**) The viability of cells treated with compounds and PQ was measured by the MTT assay. (**b**) The viability of cells treated with compounds and PQ was measured by the FDA assay. (**c**) PI-straining cells treated with compounds and PQ. Each bar represents the mean ± SEM of three independent replicates. (*) and (#), indicate, respectively, *p* < 0.05 and 0.01 versus PQ-treated cells. The difference between control and PQ-treated cells was considered significant (*p* < 0.05).

The viability of neurotoxin-treated cells was measured by both MTT and fluorescein diacetate (FDA) tests. As a result of the MTT assay, PQ-treated cell viability was 45.4% of control cells (Figure 2a), while the FDA method showed the corresponding value of 62.2% (Figure 2b). In both tests, pre-incubation with compound **1** significantly increased the PQ-treated cell viability. However, the neuroprotective effect of compound **1** measured by the FDA assay was detected at 1 and 10 μM (12% and 15%, respectively), while only a 1 μM concentration of **1** increased cell viability by 21% during the MTT assay. Compound **2** increased the PQ-treated cell viability by 20% at a concentration of 10 μM in the FDA assay and did not show any effect in the MTT assay. Acetylpenasterol (**5**) increased PQ-treated cell viability by 18% and 22% at 1 and 10 μM respectively, during the MTT test, and had a weak effect (13%) at 1 μM in the FDA assay. Compounds **3** and **4** did not show any effect on PQ-treated Neuro-2a cell viability in the tests.

Moreover, the membrane permeability of PQ-treated cells was detected by propidium iodide (PI) straining. PQ caused an increase in the number of PI-straining cells by 35% (Figure 2c), while pre-incubation with compounds **1** (10 μM), **2** (10 μM), and **4** (10 μM) statistically decreased the fluorescence of PI-straining cells by 16%, 12%, and 17%, respectively. Acetylpenasterol (**5**) decreased it by 20% and 24% at 1 and 10 μM, respectively. Thus, pre-incubation with **5** reduced membrane permeability of PQ-treated cells almost to the control value.

### 2.4. Influence on ROS Level and Mitochondrial Dysfunction in PQ-Treated Neuro-2a Cells

**Figure 3:**
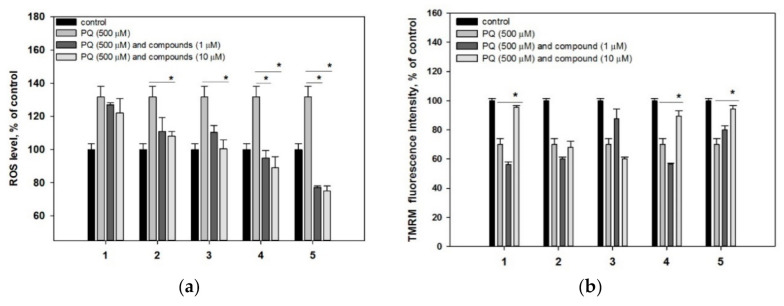
Effects of compounds **1**–**5** on ROS level (**a**) and MMP (**b**) in Neuro-2a cells after 1 h of treatment with PQ. Each bar represents the mean ± SEM of three independent replicates. (*) indicates *p* < 0.05 versus PQ-treated cells. The difference between control and PQ-treated cells was considered significant (*p* < 0.05).

The ROS level in the cells treated with PQ increased by 40% within 1 h compared to the control cells (Figure 3).

Compound **1** did not affect ROS level in PQ-treated cells. With that, pre-treating of the cells with compounds **2**–**5** resulted in a significant decrease of ROS amount by 75% and more compared to the PQ-treated cells. Among active compounds, penasterone (**4**) and acetylpenasterol (**5**) were effective at concentrations of 1 and 10 μM, while compounds **2** and **3** reduced ROS content only at a concentration of 10 μM.

The 30% decrease of tetramethylrhodamine methyl (TMRM) fluorescence after 1 h exposition of Neuro-2a cells with PQ indicates a negative effect of PQ on mitochondrial membrane potential (Figure 3b). Compounds **1**, **4**, and **5** at 10 μM diminished PQ-induced mitochondrial dysfunction and increased intensity of TMRM fluorescence by 37%, 28%, and 35%, respectively.

### 2.5. Influence on Hsp70 Level in PQ-Treated Neuro-2a Cells

**Figure 4:**
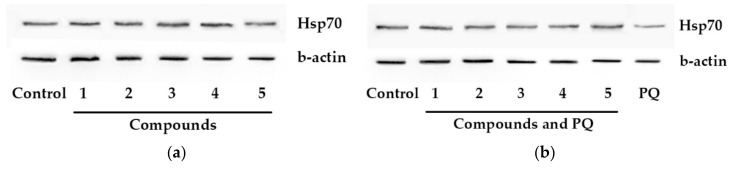

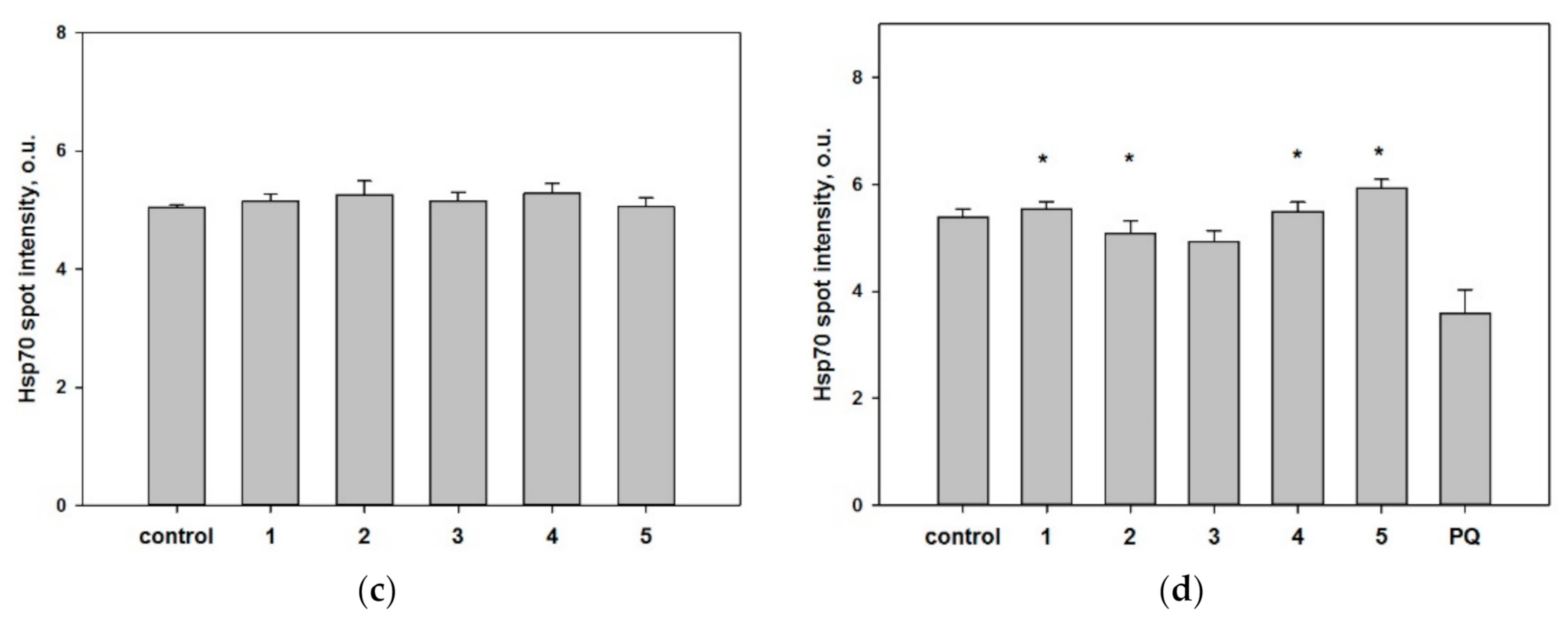
Effects of compounds **1**–**5** on Hsp70 level in Neuro-2a cells. (**a**,**c**) Neuro-2a cells were treated with compounds **1**–**5** at 10 μM for 24 h. (**b**,**d**) Neuro-2a cells were treated with compounds **1**–**5** at 10 μM for 1 h and then PQ was added for 24 h incubation. Each bar represents the mean ± SEM of three independent replicates. (*) indicates *p* < 0.05 versus PQ-treated cells. The difference between control and PQ-treated cells was considered significant (*p* < 0.05).

All investigated compounds did not affect Hsp70 level in non-treated Neuro-2a cells (Figure 4a).

PQ caused a half-decrease of Hsp70 level in Neuro-2a cells in comparison with control cells. All compounds increased Hsp70 level in PQ-treated cells to varying degrees (Figure 4b). Thus, acetylpenasterol (**5**) had the most significant effect on Hsp70 expression and raised it to the initial level. Acetylated compound **1** was more effective in this assay than non-acetylated metabolite **2** and increased Hsp70 level by 62%, while compound **2** showed an increase of 43%. Compounds **3** and **4** restored Hsp70 level by 26% and 56%, respectively.

### 2.6. Influence on Neurites Outgrowth in PQ-Treated Neuro-2a Cells

**Figure 5:**
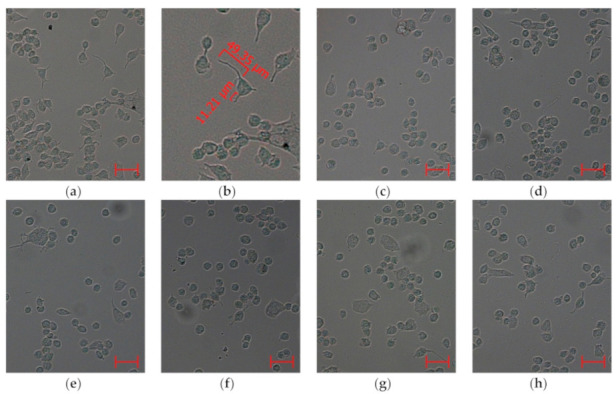
Images of Neuro-2a cells incubated without PQ (**a**,**b**), with PQ (**c**), and with investigated compounds **1** (**d**), **2** (**e**), **3** (**f**), **4** (**g**), and **5** (**h**). All compounds were used at concentrations of 10 µM. PQ were used at a concentration of 500 µM. Scale bar is 50 µm.

**Figure 6:**
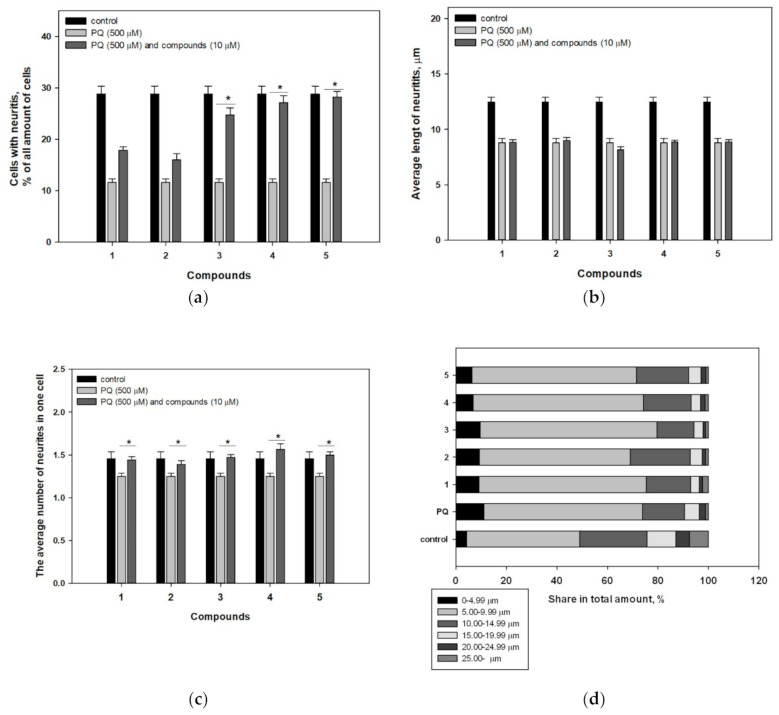
Influence of compounds **1**–**5** on neurites outgrowth in PQ-treated Neuro-2a cells. The neurites-bearing cell amount (**a**), average length of neurites (**b**), and average number of neurites (**c**) per cell, and the proportion of neurites of different lengths in the total number (**d**) are presented. Each bar represents the mean ± SEM of three independent replicates. (*) indicates *p* < 0.05 versus PQ-treated cells. The difference between control and PQ-treated cells was considered significant (*p* < 0.05).

Our results confirmed that PQ dramatically inhibited neurites outgrowth in Neuro-2a cells (Figure 5). Only 10% of PQ-treated cells had neurites, while the control contained 30% of the cells with neurites (Figure 6a). Also, the average length of neurites (Figure 6b) and number of neurites per PQ-treated cell (Figure 6c) were found to be less than control values by 29% and 14%, respectively. Moreover, the proportion of neurites of different lengths in the total number (Figure 6d) changed significantly due to the toxicity of PQ.

As a result, compounds **3**–**5** significantly increased the neurites-bearing cell amount by 113% (**3**), 134% (**4**), and 144% (**5**) respectively, compared to the number of neurites-bearing cells in the PQ-treated control. Moreover, all investigated compounds did not show an effect on the length of neurites (Figure 5), but they caused an increase in the average number of neurites per cell by 16% (**1**), 12% (**2**), 17% (**3**), 27% (**4**), and 20% (**5**). Finally, a decrease in the quantity of neurites with a length of 0–4.99 µm (6.4%) and an increase in the proportion of neurites with a length of 10.00–14.99 µm (20.7%) in the total number were observed in cells incubated with acetylpenasterol (**5**) and PQ, in comparison with PQ-treated cells (11.1% and 16.6%, respectively). Other investigated compounds had no obvious effect on the number of neurites of different lengths (Figure 6d).

## 3. Discussion

PQ-treated nerve cells are considered as one of the most useful models for studying neurotoxicity based on the generation of oxidative stress, such as PD [27]. The redox cycling of PQ on biological systems has two important implications: one is the generation of ROS and the other is the depletion of reducing equivalents (NADH, NADPH, etc.). Once the PQ has been reduced, it can then be oxidized by a molecule of oxygen and generate a superoxide molecule (O2^•−^), which occurs in neuronal cell oxidative stress and will switch to different routes that trigger cell damage in various components and initiate the activation of cellular mechanisms leading to neuron death [28]. ROS overgeneration also induces mitochondrial membrane depolarization, which plays a signal role for mitophagy as well as for mitochondria-induced apoptosis [29].

Some significant aspects of the PQ influences on the cellular system functioning have been studied using different approaches. Thus, it was reported that PQ exposure induced the most profound alterations in the pentose phosphate pathway metabolome of SK-N-SH line cells [30]. Treating of different neuronal cells (PC-12, Neuro-2a line cells, as well as human neural progenitor cells) with PQ resulted in dysregulation of miRNA profiling [31].

It was mentioned previously that Hsp70 upregulation is a histological marker of PQ-induced cell damage [32]. Nevertheless, numerous investigations confirm that pharmacological induction of Hsps expression can be considered as one of the cytoprotective mechanisms against PQ toxicity. Choi et al. reported that PEP-1–SOD fusion peptide injection protected mice against PQ by induction of Hsp70 overexpression [33]. Moreover, the activation of the heat shock protein response protects cells from α-synuclein-induced accumulation of reactive oxygen species in a metacaspase (YCA1)-dependent manner [34]. It has now been found that overexpression of Hsp70, or the induction of Hsp70 by pharmacological agents, protects against neurotoxicity in in vivo and in vitro cellular, yeast, and fly models of PD [35,36].

The PQ influences on genes’ expression were investigated earlier [5]. Lenzken et al. described that genes of “Cellular Growth and Proliferation”, “Cell Death”, and “Cell Cycle” categories were expressed differently in the PQ-treated SH-SY5Y cells compared with the controls. Also, the downregulation of the neurotrophin receptor NTRK1 in PQ-treated cells was shown by Western blot analysis [5]. Finally, the direct influence of PQ treatment on neurite outgrowth was investigated by using neuronal precursor Lund human mesencephalic LUHMES cells. This study confirmed that PQ acts as a specific neurite growth inhibitor [37].

Based on the above-mentioned mechanisms of PQ neurotoxicity, it can be assumed that biologically active compounds that act as antioxidants and capture ROS and mitochondrial dysfunction, as well as compounds that enhance the expression of Hsps and stimulate the growth of neurites, may be able to protect neuronal cells from the toxic effects of neurotoxin and defend them from the death.

Indeed, among the natural substances analyzed in the present study, a direct correlation between an increase in the viability of cells treated with PQ and a decrease in the level of ROS in these cells was observed for compounds **2**, **4**, and **5**. For **4** and **5**, these effects go with the considerable diminishing of the mitochondrial membrane depolarization. Compound **1** showed a good protective effect on PQ-treated cell viability, while it did not influence on ROS level in the cells. In contrast, compound **3** did not demonstrate any influence on viability of PQ-treated cells but it significantly decreased intracellular ROS level. At the same time, none of the tested substances showed antioxidant properties in the DPPH radical scavenger assay. In this regard, we assumed that the observed effects of **2**–**5** on the ROS production were due to other properties of these compounds, for example, their ability to affect the activity of a number of enzymes that maintain the ROS balance in the cell. The fact that the results of MTT and FDA studies of the viability of cells treated with the test compounds were different, confirmed this assumption. Because, in the MTT assay case, the activity of NAD(P)H-dependent oxidoreductases that reduce the tetrazolium dye to formazan is determined, while the FDA test evaluates the activity of the cytoplasmic nonspecific esterase. We have observed previously that oxygenated sterols from the marine sponge *Inflatella* sp. caused overestimation during the MTT assay and demonstrated higher cell viability rates in comparison with the results of the FDA test [38]. Moreover, some lanostane triterpenoids were shown to induce NAD(P)H quinone oxidoreductase activity [39]. Since acetylpenasterol (**5**) was more active in the MTT assay than in the FDA investigation, we believe that it can induce intracellular antioxidant factors significant for protection against PQ-induced toxicity, analogously to some other terpenoid compounds, including the recently reported 3*β*-angeloyloxy-8*β*,10*β*-dihydroxyeremophila-7(11)-en-12,8*α*-lactone. This eremophilane sesquiterpenoid from *Farfugium japonicum* was shown to protect neuronal cells against PQ-induced oxidative injury and toxicity by activating some antioxidative factors, including superoxide dismutase and reduced glutathione [40]. Moreover, a cytoprotective effect of compounds **1**, **2**, **4**, and **5** against PQ toxicity was detected by PI-straining, which indicates necrosis or cell membrane permeabilization.

The obtained results also demonstrated the protective effect of investigated triterpenoids **1**–**5** accompanied with an increasing Hsp70 intracellular level in Neuro-2a cells. Among others, compounds **1** and **5** were the most effective. As these data correlate with their ability to increase PQ-treated cell viability, and it is possible that the upregulation of Hsp70 protein expression plays a significant role in the neuroprotection mechanism of these triterpenoids.

One more promising property of the analyzed metabolites from *Penares* sp. was revealed during the research. Namely, we have discovered that all investigated triterpenoids caused an increase of the average number of neurites per cell. Moreover, pre-treatment with compounds **3**–**5** significantly increased the number of neurite-bearing cells. It is known that some lanostane triterpenoids exhibit neuritogenic activity. Thus, ganoleucoins Q and R showed protective effects against the H_2_O_2_-induced PC12 cell damage and enhanced neurite outgrowth in non-treated PC12 cells [41]. Also, cucurbitacin B exhibited significant nerve growth factor (NGF)-mimic or NGF-enhancing activity in PC12 and primary neuron cells [42]. Nevertheless, lanostane triterpenoids have not been previously described as neuritogenic agents in PQ or another neurotoxin-induced PD model.

Thereby, the five studied triterpenoids demonstrated neuroprotective effects against PQ-induced toxicity by antioxidant-dependent as well as antioxidant-independent pathways. All compounds increased the number of neurites, and some of them, 29-*nor*-penasterone (**3**), penasterone (**4**), and acetylpenasterol (**5**), increased the number of neurite-bearing cells in the PQ-treated cell population. Moreover, acetylpenasterol (**5**) increased the viability of PQ-treated cells, which was shown by two independent assays. The influence of 3*β*-acetoxy-7*β*,8*β*-epoxy-5*α*-lanost-24-en-30,9α-olide (**1**) on cell viability was more perceptible but this compound did not show a statistical effect on the number of neurite-bearing PQ-treated cells. A comparison of **1** and **5** activities in the corresponding tests demonstrated that **1** did not affect the ROS level in the cells, whereas **5** significantly reduced it. In other tests, the effects of **1** and **5** were comparable.

The analysis of structure–activity relationships revealed a significance of 3-acetoxy substituent along with the gem-dimethyl group at C-4 for neuroprotective activity of investigated triterpenoinds. Indeed, the presence of the mentioned features in structures **1** and **5** correlate with higher activity of the compounds in most tests. Moreover, the fact that metabolite **5** was more active than compound **1** could indicate the importance of the free carboxy group at C-17 for neuroprotective effect.

## 4. Materials and Methods

### 4.1. Compounds

The isolation of metabolites **1**–**5** from marine sponge *Penares* sp. and their structural investigation have been reported previously [25]. Before the bioassay, all compounds were re-chromatographed using described high pressure liquid chromatography (HPLC) procedures [25] and their chemical purity was confirmed by high resolution electrospray ionization mass-spectrometry (HRESIMS). Chemical structures of studied compounds are presented in Figure 1. All compounds were dissolved in dimethylsulfoxid (100%) at a concentration of 10 mM. These solutions were used to obtain the required concentration of compounds in the cell suspension so that the concentration of DMSO in the cell suspension did not exceed 1%.

### 4.2. DPPH Radical Scavenging Assay

The 2,2-diphenyl-1-picryl-hydrazyl-hydrate (DPPH) radical scavenging activity was tested as described previously [43]. The compounds were dissolved in MeOH, and the solutions (120 µL) were dispensed into wells of a 96-well microplate. The DPPH (Sigma-Aldrich, Steinheim, Germany) was dissolved in MeOH at a concentration of 7.5 × 10^−3^ M and the solution (30 µL) was added to each well. The concentrations of tested compounds in the mixtures were 10 and 100 µM. Ascorbic acid was used as a positive control. The mixtures were shaken and left for 30 min. The absorbance of the resulting solutions was measured at λ = 520 nm with a MultiscanFC plate reader (Thermo Scientific, Waltham, MA, USA). The concentration scavenging 50% of the DPPH radical (EC_50_) was calculated for each compound.

### 4.3. Neuro-2a Cell Culture

The cells of mouse neuroblastoma cell line Neuro-2a (ATCC^®^ CCL-131™; American Type Culture Collection, Manassas, VA, USA) were cultured in Dulbecco’s modified Eagle’s medium (DMEM) containing 10% fetal bovine serum (Biolot, St. Petersburg, Russia) and 1% penicillin/streptomycin (Biolot, St. Petersburg, Russia) at 37 °C in a humidified atmosphere with 5% (*v*/*v*) CO_2_. Initially, cells were incubated in cultural flasks until sub-confluent (~80%). For testing, Neuro-2a cells were seeded in 96- or 6-well plates and experiments were started after 24 h.

### 4.4. MTT Cell Viability Assay

The cells (1 × 10^4^ cells/well of a 96-well plate) were incubated with different concentrations of studied compounds during 24 h. After that, cell viability was determined by the MTT (3-(4,5-dimethylthiazol-2-yl)-2,5-diphenyltetrazolium bromide) method according to the manufacturer’s instructions (Sigma-Aldrich, St. Louis, MO, USA). Absorbance of the converted formazan was measured using a Multiskan FC microplate photometer (Thermo Scientific, Waltham, MA, USA) at λ = 570 nm. The results were presented as percent of control data, and concentration of cell viability inhibition on 50% (IC_50_) was calculated.

### 4.5. FDA Cell Viability Assay

A stock solution of the fluorescein diacetate (FDA) (Sigma-Aldrich, St. Louis, MO, USA) in DMSO (1 mg/mL) was prepared. The cells (1 × 10^4^ cells/well) were seeded in black 96-well plates before testing. After incubation of the cells with compounds and PQ during 24 h, FDA solution (50 μg/mL) was added to each well and the plate was incubated in the dark at 37 °C for 15 min. Cells were washed with phosphate buffer saline (PBS) (BioloT, S.-Peterburg, Russia) and fluorescence was measured with a Fluoroskan Ascent plate reader (Thermo Labsystems, Helsinki, Finland) at λ_ex_ = 485 nm and λ_em_ = 518 nm. All manipulations with fluorescent probes were carried out in a darkened room. Cell viability was expressed as the percent of control.

### 4.6. Propidium Iodide Straining

The cells (1 × 10^4^ cells/well) were seeded in black 96-well plates before testing. After incubation of the cells with compounds and PQ during 24 h, PI solution (10 μg/mL) was added to each well and the plate was incubated in the dark at 37 °C for 10 min. Cells were washed with phosphate buffer saline (PBS) (BioloT, St.Peterburg, Russia) and fluorescence was measured with a Fluoroskan Ascent plate reader (Thermo Labsystems, Helsinki, Finland) at λ_ex_ = 485 nm and λ_em_ = 518 nm. All manipulations with fluorescent probes were carried out in a darkened room. The results were presented as percent of positive control data.

### 4.7. Paraquat-Induced Neurotoxicity

The cells (1 × 10^4^ cells/well of a 96-well plate) were treated with studied compounds at concentrations of 1 and 10 µM for 1 h and then 500 µM of PQ (Sigma-Aldrich, St. Louis, MO, USA) was added to the neuroblastoma cells. Cells incubated without PQ and compounds and with PQ alone were used as positive and negative controls, respectively. The viability of cells was measured after 24 h using MTT and FDA methods. The results were presented as percent of positive control data.

### 4.8. Reactive Oxygen Species (ROS) Level Analysis

The cells (1 × 10^4^ cells/well of a 96-well plate) were incubated with compound solutions (1 and 10 µM) during 1 h. Then, PQ was added to cell suspension to a resulting concentration of 500 µM for incubation during 1 h. Cells incubated without PQ and compounds and with PQ alone were used as positive and negative controls, respectively. The 20 µL of 2,7-dichlorodihydrofluorescein diacetate solution (H_2_DCFDA, Molecular Probes, Eugene, OR, USA) was added to each well (10 µM, final concentration) and the plate was incubated for an additional 10 min at 37 °C. The intensity of dichlorofluorescin fluorescence was measured with a PHERAstar FS plate reader (BMG Labtech, Ortenberg, Germany) at λ_ex_ = 485 nm and λ_em_ = 518 nm. The data were processed by MARS Data Analysis v. 3.01R2 (BMG Labtech, Ortenberg, Germany). The results were presented as percent of positive control data.

### 4.9. Mitochondrial Membtane Potential (MMP) Detection

The cells (1 × 10^4^ cells/well of a 96-well plate) were incubated with compound solutions (1 and 10 µM) during 1 h. Then, PQ was added to cell suspension to a resulting concentration of 500 µM for incubation during 1 h. Cells incubated without PQ and compounds and with PQ alone were used as positive and negative controls, respectively. The tetramethylrhodamine methyl (TMRM) (Sigma-Aldrich, St. Louis, MO, USA) solution at 500 nM was added in each well and cells were incubated for 30 min at 37 °C. The intensity of fluorescence was measured with a PHERAstar FS plate reader (BMG Labtech, Ortenberg, Germany) at λ_ex_ = 540 nm and λ_em_ = 590 nm. The data were processed by MARS Data Analysis v. 3.01R2 (BMG Labtech, Ortenberg, Germany). The results were presented as percent of positive control data.

### 4.10. Hsp70 Level Analysis

The Hsp70 expression was detected by Western blot analysis. Cell suspensions (1.6 × 10^5^ cells/well of a 6-well plate) were seeded and treated with studied compounds at a concentration of 10 µM for 1 h. After that, PQ (500 µM) was added and cells were incubated during 24 h. Cells incubated without PQ and compounds and with PQ alone were used as positive and negative controls, respectively. Cells were washed by cold PBS (BioloT, Russia) twice and lysed by radioimmunoprecipitation assay (RIPA) buffer (BioloT, St. Peterburg, Russia). The amount of proteins was measured by the Bradford method and equal samples (25 µg) were prepared using sample buffer. Proteins were separated with sodium dodecyl sulphate (SDS) electrophoresis in 10% polyacrylamide gel. The electrophoretically separated proteins were transferred onto the polyvinylidene difluoride (PVDF) membrane Whatman (Sigma-Aldrich, St. Louis, MO, USA) using a semi-dry transfer apparatus (Helicon, Moscow, Russia). The Hsp70 protein zone was revealed using specific primary monoclonal antibodies against Hsp70 BRM-22a (Abcam, Cambridge, UK) in dilution of 1:5000 and secondary antibodies were conjugated with horseradish peroxidase in a dilution of 1:10,000 (Sigma-Aldrich, St. Louis, MO, USA). b-Actin zones were revealed using specific monoclonal antibodies (Abcam, Cambridge, UK) and used as a loading control. The peroxidase reaction was visualized by an enhanced chemiluminescence (ECL) kit according to the instructions of the manufacturer (Sigma-Aldrich, St. Louis, MO, USA) using the VersaDoc Imaging System (Bio-Rad, Hercules, CA, USA).

### 4.11. Neurite Outgrowth Estimation

Neurite outgrowth was studied as described by Li et al. [44]. Neuro-2a cells were seeded in 6-well plates (1.6 × 10^5^ cells/well) in DMEM medium containing 10% fetal bovine serum (Biolot, St. Peterburg, Russia) and 1% penicillin/streptomycin (Invitrogen, Carlsbad, CA, USA). The next day, this growth media was changed with serum-free DMEM media containing 1% penicillin/streptomycin, and cells were treated with studied compounds at a concentration of 10 µM for 1 h. After that, PQ (500 µM) was added and cells were incubated during 24 h. Cells incubated without PQ and compounds and with PQ alone were used as positive and negative controls, respectively.

For each well, approximately 10 images were acquired randomly by scanning the wells from left to right and top to bottom using the AxioImager A1 microscope (CarlZeiss, Oberkochen, Germany). A total of ten random fields (near 350 cells in each) for each experimental group were scored for neurite outgrowth. The number of neurite-bearing cells and lengths of the individual neurites for each cell were measured using the AxioVision 4.7.1 software (CarlZeiss, Oberkochen, Germany). Length was defined as the straight-line distance from the tip of the neurite to the junction between the cell body and neurite base. In the case of branched neurites, the length of the longest branch was measured from the tip of the neurite to the cell body. In each experimental group, the average length of neurites was calculated, as well as the average number of neurites per one cell.

### 4.12. Data Evaluation

All data were obtained in three independent replicates and calculated values were expressed as mean ± standard error of mean (SEM). Student’s t-test was performed using SigmaPlot 14.0 (Systat Software Inc., San Jose, CA, USA) to determine statistical significance.

## 5. Conclusions

To summarize the obtained results, we can conclude that among the studied lanostane triterpenoids, acethylpenasterol (**5**) is the most promising candidate for further investigation of its neuroprotective potential.

## Figures and Tables

**Table 1:** Cytotoxic and radical scavenging activities of compounds **1**–**5**.

Compound	Cytotoxicity (MTT Assay)		DPPH Radical Scavenging
	IC_50_, μM	% of Control at 10 μM	EC_50_, μM
**1**	85.7 ± 2.4	92.1 ± 7.1	>100
**2**	>100	96.9 ± 1.1	>100
**3**	>100	91.1 ± 4.1	>100
**4**	>100	95.3 ± 2.7	>100
**5**	>100	97.9 ± 3.3	>100
Ascorbic acid	-		20.5 ± 1.5
